# Human-T-Cell-Selective Fluorescent Probe

**DOI:** 10.3390/cells11182836

**Published:** 2022-09-11

**Authors:** Min Gao, Young-Tae Chang

**Affiliations:** 1 Center for Self-Assembly and Complexity, Institute for Basic Science (IBS), Pohang 37673, Korea; 2 Department of Chemistry, Pohang University of Science and Technology (POSTECH), Pohang 37673, Korea

**Keywords:** cell recognition, fluorescent probes, high-throughput screening, mitochondria

## Abstract

The identification of T and B lymphocytes has relied on using antibodies against different biomarkers as the gold standard. Emerging small molecule-based fluorescent probes have the potential to replace antibodies. Herein, we report the first human-T-cell-selective fluorescent probe, Mito thermo yellow (MTY), achieving the live T cells’ distinction from B cells, which was previously impossible without the help of antibodies. The unexpected cell selectivity of MTY is attributed to the higher mitochondria mass and membrane potential of T cells over B cells. This study enriches the toolbox for live cell distinction from complex cell communities.

## 1. Introduction

T and B cells, two different types of lymphocytes, carry out the adaptive immune response: humoral immune responses and cell-mediated immune responses [1]. B cells are activated to secrete antibodies for humoral immune response, whereas T cells are able to recognize antigens and execute effector functions for the regulation of cellular immune response [2,3,4]. T cells can be further divided into three subsets: CD4^+^ helper T cells, CD8^+^ cytotoxic T cells and regulatory T cells, based on their expression of certain surface molecules and different functions in controlling and shaping the immune response [5,6,7].

**Figure 1:**
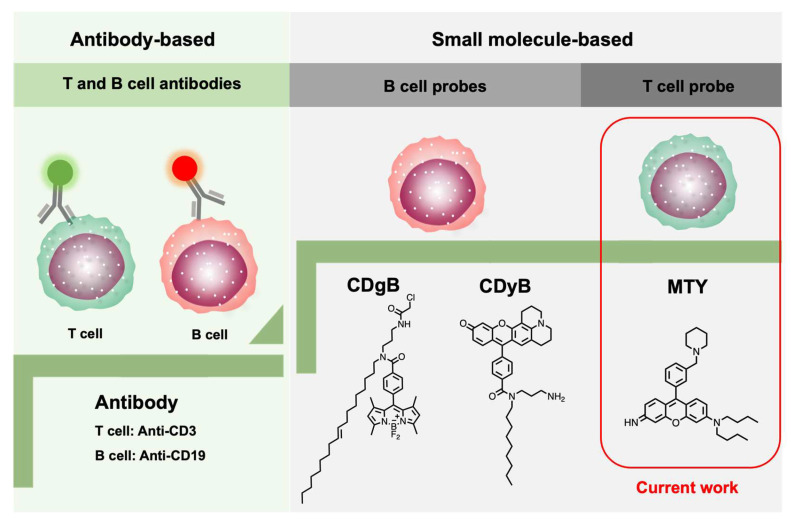
Development progress of T- and B-cell identification.

T and B cells are derived from the same multipotent hematopoietic stem cell and look identical to one another. Conventionally, cell-surface-targeting anti-CD (cluster of designation) antibodies have been used to discriminate different cell types, as different cells may express unique biomarkers [8,9]. Emerging small molecule-based fluorescent probes have the potential to replace the antibodies. Recently, two fluorescent probes, CDgB and CDyB, for B cells’ distinction from T cells, have been developed by our group using unbiased screening with the Diversity-Oriented Fluorescence Library (DOFL), breaking the conventional method (Figure 1) [10,11]. DOFL is a toolbox for cell-type distinction by combinatorial screening [12,13]. Meanwhile, some fluorescent probes only targeting to cytotoxic T lymphocytes (CTLs) have been reported and they are also nano-materials [14,15]. So far, the whole T-cell-selective probe is still empty. Therefore, it is crucial and timely to develop a T-cell-selective fluorescent probe, especially a freely cell-permeable small molecule probe.

Herein, we developed the first human-T-cell-specific fluorescent probe, Mito thermo yellow (MTY), based on a high-throughput screening method with the DOFL (Figure 1). MTY could accurately distinguish the T cells from B cells. The high mitochondria mass and membrane potential of T cells contributed to the selectivity of MTY for T cells.

## 2. Materials and Methods

### 2.1. Compound Information

Stock solution of probe MTY (1.0 mM) was prepared in HPLC-grade DMSO and stored at 4 °C in darkness. The maximum absorbance and emission wavelength were 542 and 564 nm, respectively, in HEPES 20 mM (pH 7.4).

### 2.2. Blood

The peripheral blood samples from healthy donors were obtained by S Pohang hospital (Pohang, Korea). The Hospital’s Ethics Committee and Research Ethics Advisory Committee approved all experimental procedures (PSSH0475-202104-BR-001). Written informed consent was obtained from all donors prior to participation in the study. The blood was drawn from the median cubital vein in the antecubital fossa.

### 2.3. Preparation of White Blood Cell

White blood cells (WBCs) were obtained after lysis of red blood cells (RBCs) using RBC lysis buffer (Thermo Fisher Scientific, Rockford, IL, USA). The white blood cell was suspended in the media containing Roswell Park Memorial Institute (RPMI) 1640 Medium (Gibco, Invitrogen, Carlsbad, CA, USA), 10% Fetal Bovine Serum (FBS, Gibco, Invitrogen, Carlsbad, CA, USA) and 1% Penicillin Streptomycin (Thermo Fisher Scientific, Rockford, IL, USA).

### 2.4. High-Throughput Screening

For high-throughput screening, white blood cells isolated from human blood were resuspended in media (RPMI 1640 + 10% FBS + 1% PS) to make the density 2.5 × 10^6^/mL. Then the cells were incubated with 1 μM of library compounds. After 1 h, the samples were read by flow cytometry. The screening was conducted through three independent tests.

### 2.5. Human T and B Cells’ Isolation

The white blood cells were passed through 40 μm cell strainers to obtain single-cell suspensions. For human T and B cell isolation, the T cells Isolation Kit (BD™ IMag T Lymphocyte Enrichment Set–DM, Franklin Lakes, NJ, USA) and B cells Isolation Kit (BD™ IMag B Lymphocyte Enrichment Set–DM, Franklin Lakes, NJ, USA) were used. These BD™ IMag Enrichment set was used for the negative selection.

### 2.6. Fluorescence Microscopy and Flow Cytometry Analysis

Fluorescence image analysis was conducted on an Operetta CLS™ High-Content Analysis System (PerkinElmer, Waltham, MA, USA). Flow cytometry was conducted on an S3e cell sorter (Bio-Rad, Hercules, CA, USA). Data analysis was performed using BD FlowJo^TM^ v10 software (Franklin Lakes, NJ, USA).

## 3. Results and Discussion

### 3.1. Development of Human-T-Cell-Selective Probe

To develop a human-T-cell-selective probe, thousands of compounds were collected and unbiased cell-based screening was conducted. In peripheral blood, T cells account for approximately 70% of all lymphocytes, while B cells and NK cells account for 23% and 7% of all lymphocytes, respectively [16]. Therefore, peripheral blood from healthy volunteers was chosen as the screening system. For the high-throughput screening platform, white blood cells obtained from peripheral blood were incubated with library compounds for 1 h. Then lymphocytes were analyzed using flow cytometry by Forward Scatter (FSC) versus Side Scatter (SSC) plot gating. After the fluorescent compound addition, if the lymphocytes showed the separated populations, the compound was picked as the hit candidate. Then, human T and B cells were isolated to check the selectivity of this compound.

**Figure 2:**
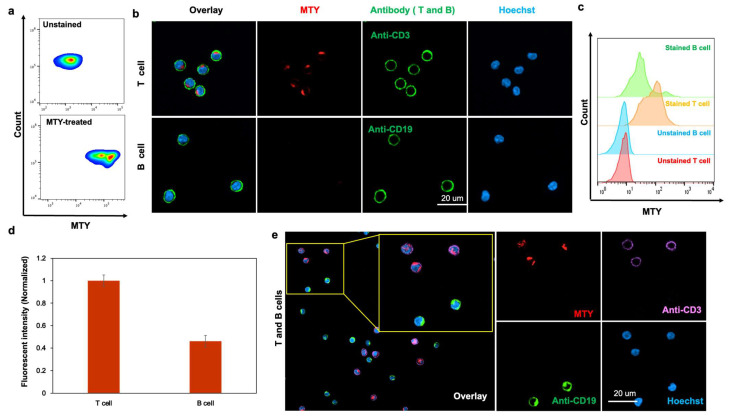
Selectivity of MTY in human T and B cells. (**a**) MTY performance in the lymphocyte of blood. The WBCs were incubated with MTY (1 µM) for 1 h. (**b**,**c**) Selectivity in isolated human T and B cells. T cells were colocalized with MTY and CD3 antibody. B cells were colocalized with MTY and CD19 antibody. Fluorescence images (**b**) and flow cytometry (**c**) were taken after 1 h incubation with MTY (100 nM) and 30 min with antibody. (**d**) Quantitative comparison of fluorescent intensity by flow cytometry. Fluorescent intensity in Figure 3c was normalized and represented as histogram. (**e**) Selectivity of MTY in mixed T and B cells. T cells and B cells were randomly mixed, and then treated with MTY (100 nM), Hoechst (1 µg/mL), CD3 antibody and CD19 antibody.

Through three independent screenings, MTY was chosen as the only candidate for the T-cell probe (Figure 1). MTY showed higher fluorescence in isolated T cells than in B cells, indicating its favorable selectivity for T cells by flow cytometry and fluorescent imaging (Figure 2a–d). To further determine the ability of MTY for T cell distinction, T cells and B cells were mixed together, then MTY, anti-CD3 and anti-CD19 were added to the cells at the same time. CD3 and CD19 are standard T and B cell markers, respectively. Expectedly, the fluorescence signal of MTY and anti-CD3 appeared in the same cell, whereas the MTY signal was not shown in the anti-CD19-stained cells, indicating that MTY was able to clearly distinguish the T cells rather than B cells in the mixture of cells (Figure 2e).

### 3.2. Localization of MTY

**Figure 3:**
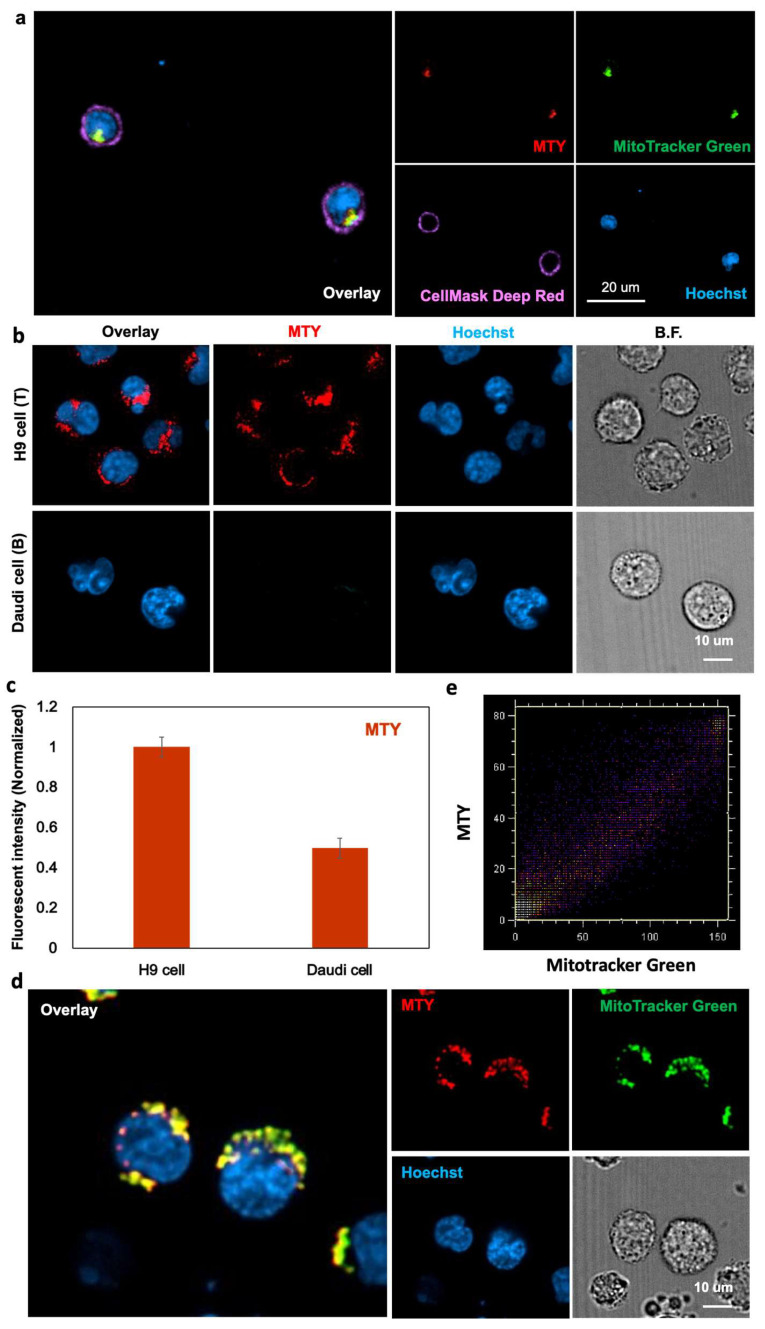
Localization of MTY. (**a**) Localization of MTY in human primary T cells. T cells were treated with MTY (100 nM), Mitotracker Green (200 nM), Cellmask Deep Red (5 ug/mL) and Hoechst (1 µg/mL) for 1 h, 30 min, 30 min and 10 min, respectively. (**b**,**c**) Selectivity of MTY in the H9 and Daudi cells. Fluorescence images (**b**) and flow cytometry (**c**) were obtained after 1 h incubation with MTY (100 nM). Fluorescent intensity evaluated by flow cytometry was normalized and represented as histogram. (**d**) Localization of MTY in H9 cells. H9 cells were treated with MTY (100 nM), Mitotracker Green (200 nM) and Hoechst33342 (1 µg/mL) for 1 h, 30 min and 10 min, respectively. (**e**) Display of the colocalization areas of the red and green channels in (**d**).

At the subcellular level, MTY overlapped with Mitotracker Green very well in the T cells, which demonstrated that MTY localized in the mitochondria of T cells (Figure 3a). Due to the small size of primary T cells, a typical T cell line, the H9 cells, was chosen with its larger size to clearly demonstrate MTY localization, because the selectivity of MTY remained unchanged in T and B cell lines, H9 cells and Daudi cells (Figure 3b,c). Therefore, H9 cells can be used as representative cells for localization imaging. In H9 cells, MTY stained the mitochondria clearly due to its fluorescence and merged well with Mitotracker Green (Figure 3d). The Pearson’s coefficient of MTY and Mitotracker Green was 0.949. The intensity distribution of MTY and Mitotracker Green showed a high correlated plot by the color–pair intensity correlation analysis (Figure 3e). The localization of MTY in the mitochondria was consistent with the previous study [17].

### 3.3. Selectivity Mechanism

**Figure 4:**
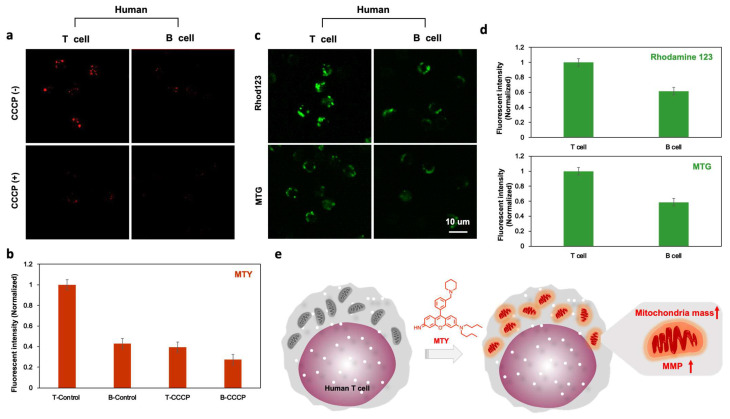
Mechanism for the selectivity of MTY. (**a**,**b**) MMP effect on the fluorescence of MTY in human T and B cells. The cells were first treated with CCCP (50 µM) for 30 min, and then MTY (100 nM) was added for 1 h. Fluorescent intensity evaluated by flow cytometry was normalized and represented as histogram. (**c**,**d**) The fluorescence of MTG (200 uM) and Rhod123 (100 uM) in human T and B cells. The fluorescence images were taken after 30 min incubation of MTG and Rhod123. Fluorescent intensity evaluated by flow cytometry was normalized and represented as histogram. (**e**) The proposed staining mechanism of MTY.

Once MTY was identified as a T cell probe, the next step was to investigate the selective staining mechanism of this probe towards T cells. A mitochondria-localized dye was inspired by MTY, and the factors related to mitochondria, such as mitochondria membrane potential (MMP) and mitochondria mass, should be considered. CCCP is a mitochondrial oxidative phosphorylation uncoupler that increases membrane permeability to protons and dissipates this proton gradient, leading to a disruption in the mitochondrial membrane potential [17]. After depolarization of MMP with CCCP in human T and B cells, the fluorescence of MTY was dramatically reduced, more in T cells than B cells, which demonstrated that T cells contained higher MMP than B cells and MTY was MMP-dependent (Figure 4a,b). To confirm it, rhodamine 123 was used to assess the difference in MMP between T and B cells. The MMP in human T cells was higher than that in human B cells, which was consistent with the previous study [18]. Therefore, the higher MMP of T cells contributed to the selectivity of MTY to T cells (Figure 4c,d).

Interestingly, T cells still showed slightly stronger fluorescence of MTY than B cells after CCCP addition (Figure 4a), which means in addition to MMP, mitochondria mass may play a role toward the selectivity of MTY for T cells. To confirm this hypothesis, the mitochondrial mass of T and B cells was checked by Mitotracker Green. Consistently, human T cells had higher mitochondria mass than human B cells (Figure 4b), which is also matched with the previous study [18]. Therefore, the higher mitochondria mass of T cells also promoted the selectivity of MTY. Altogether, human T cells possess higher mitochondria mass and membrane potential, which drove the selectivity of MTY for human T cells (Figure 4e). Interestingly, these mitochondrial differences between T and B cells were already reported [18], but they has not been actively used for selective probe development. In our study, through unbiased screening, the discovered probe MTY resummoned the basis of the cell selectivity.

In this study, our main focus was to develop a fluorescent probe selective for T cells over B cells, by the DOFL approach. This method requires no knowledge about the biomarker of the cell, which allows us to explore a wide range of new targets for live cell distinction. With this study, we were able to distinguish T cells over B cells by higher MMP and mitochondria mass, which is a new discovery. However, we understand the limitation that this probe only works between T and B cells. For further applications in complex biological systems, modifications and optimizations of the compound structure by structure–activity relationship (SAR) study might be necessary to discriminate the subtype of T cells. With the low interference of T cell function, we expect MTY will be an excellent probe to monitor the cell activities and explore various functions in live cells under more natural condition.

## 4. Conclusions

Here, we presented the first T-cell-selective probe, MTY, by high-throughput screening and elucidated its selectivity mechanism. Compared with B cells from peripheral blood, this probe showed high selectivity for T cells. MTY was localized in the mitochondria specifically. Inspired by the localization of MTY, the high mitochondria mass and MMP of T cells were checked and confirmed to contribute to the high selectivity of MTY. Since this is the first fluorescent probe for T cells, it fills the gap in distinguishing T cells based on small molecules. Together with B-cell probes, it enriches the molecular toolbox for live cell distinction.

## Data Availability

The data presented in this study are available on request from the corresponding authors.
